# Retinal thinning of inner sub-layers is associated with cortical atrophy in a mouse model of Alzheimer’s disease: a longitudinal multimodal in vivo study

**DOI:** 10.1186/s13195-019-0542-8

**Published:** 2019-11-13

**Authors:** Samuel Chiquita, Elisa J. Campos, João Castelhano, Mário Ribeiro, José Sereno, Paula I. Moreira, Miguel Castelo-Branco, António Francisco Ambrósio

**Affiliations:** 1 Coimbra Institute for Clinical and Biomedical Research (iCBR), Faculty of Medicine, University of Coimbra, 3000-548 Coimbra, Portugal; 2 CNC.IBILI Consortium, University of Coimbra, 3004-504 Coimbra, Portugal; 3 Coimbra Institute for Biomedical Imaging and Translational Research (CIBIT), University of Coimbra, 3000-548 Coimbra, Portugal; 4 Institute for Nuclear Sciences Applied to Health (ICNAS), University of Coimbra, 3000-548 Coimbra, Portugal; 5 Center for Neuroscience and Cell Biology (CNC), University of Coimbra, 3004-517 Coimbra, Portugal; 6 Institute of Physiology, Faculty of Medicine, University of Coimbra, 3004-517 Coimbra, Portugal

**Keywords:** Alzheimer’s disease, 3×Tg-AD mouse model, Retina, Brain

## Abstract

**Background:**

It has been claimed that the retina can be used as a window to study brain disorders. However, concerning Alzheimer’s disease (AD), it still remains controversial whether changes occurring in the brain and retina are associated. We aim to understand when changes start appearing in the retina and brain, how changes progress, and if they are correlated.

**Methods:**

We carried out a unique longitudinal study, at 4, 8, 12, and 16 months of age, in a triple transgenic mouse model of AD (3×Tg-AD), which mimics pathological and neurobehavioral features of AD, as we have already shown. Retinal structure and physiology were evaluated in vivo using optical coherence tomography and electroretinography. Brain visual cortex structure was evaluated in vivo using magnetic resonance imaging.

**Results:**

The retinal thickness of 3×Tg-AD decreased, at all time points, except for the outer nuclear layer, where the opposite alteration was observed. Amplitudes in scotopic and photopic responses were increased throughout the study. Similarly, higher amplitude and lower phase values were observed in the photopic flicker response. No differences were found in the activity of retinal ganglion cells. Visual cortex gray matter volume was significantly reduced.

**Conclusions:**

Our results show that this animal model shows similar neural changes in the retina and brain visual cortex, i.e., retinal and brain thinning. Moreover, since similar changes occur in the retina and brain visual cortex, these observations support the possibility of using the eye as an additional tool (noninvasive) for early AD diagnosis and therapeutic monitoring.

## Background

The concept of “the retina as a window to the brain” has emerged with possible implications in various pathologies, such as Alzheimer’s disease (AD), Parkinson’s disease and multiple sclerosis [1]. In AD, it is still difficult to identify subclinical biomarkers that can predict the subsequent appearance of symptoms [2]. Moreover, in order to have a definitive diagnosis of AD, there is the need to identify amyloid β (Aβ) plaques and neurofibrillary tangles post-mortem [3–5]. To achieve better and earlier treatments for AD, it is crucial to target early diagnosis. Thus, there is an urgent need to identify new biomarkers that can reliably help diagnose early AD onset [6].

Visual alterations have been detected in AD patients, and those alterations can be associated with structural and functional changes in the retina [7–9]. Retinal imaging and electrophysiology can be used to evaluate structural and functional abnormalities in the retina, a more accessible structure for a potential early AD detection [10, 11]. The eye enables to perform noninvasive, inexpensive and in vivo tests, which is often not the case concerning the brain. In particular, optical coherence tomography (OCT) measurements have shown that there is a thinning of the retina in AD patients [12–16]. Actually, manifestations in AD, Parkinson’s disease and multiple sclerosis have been reported with the aid of OCT quantifications [17–21]. Retinal nerve fiber layer (RNFL) thickness reduction has been observed in AD patients [8, 21–25]. However, other authors have claimed that there are no differences in RNFL thickness between mild cognitive impairment or AD patients and control groups [26–29]. Further studies in AD patients also showed the reduction of ganglion cell layer (GCL) [18, 30, 31] and inner plexiform layer (IPL) thickness [32–34], as well as cortical atrophy, namely the decrease in the volume of visual cortex [35, 36]. Recently, the existence of Aβ plaques in the retina was reported in several AD animals models [14], and in both post-mortem retinas of advanced and early stage AD patients [37, 38] and, using curcumin-based fundus imaging, in AD patients [39].

Aiming to clarify the controversy related with the possible use of the eye as a window to the brain in AD, longitudinal studies with large cohorts need to be carried out. Mouse models of AD offer the possibility of performing longitudinal studies on a much shorter timescale than in AD patients. There are several studies designed to assess retinal structural and functional changes in AD murine models [5, 40–46]. Conversely, there are only a few studies that assess the pathophysiology of AD in animal models simultaneously in the retina and brain, trying to establish possible correlations between these structures [5, 47–51]. These studies assessed only few structural and functional parameters, without expressing the inner sub-layers detail, Therefore, we aimed to get further insight into the pathophysiology of AD and, particularly, to understand if changes in the retina correlate with changes in the visual cortex. To achieve this goal, we performed a unique and novel longitudinal study using the 3×Tg-AD mouse model. We previously showed that 3×Tg-AD mice have recognition memory impairment, early hippocampal structural loss, increased Aβ and hyperphosphorylated tau, and decreased levels of taurine [52]. In the present study, we assessed simultaneously the retinal and visual cortex alterations, in vivo, at four different time points: 4, 8, 12 and 16 months of age. This is, thus, an approach much more relevant for clinical setting. Furthermore, the combination of structural and functional changes, either in the retina and brain regions, such as the visual cortex, might be used as biomarker for early AD diagnosis.

## Methods

### Animals

Experiments were performed in male 3×Tg-AD mice harboring three human mutant genes, presenilin-1 (PS1_M146V_), amyloid precursor protein (APP_SWE_) and tau (tau_P301L_), which develop the pathological hallmarks of AD in an age-dependent manner [53, 54], and in gender- and age-matched WT animals (C57BL6/129S background). The 3×Tg-AD line was originally generated by co-microinjection of human APP_K670M/N671L_ and tau_P301L_ transgenes, under the control of the Thy 1.2 promoter, into mutant PS1_M146V_ knock-in mice [54]. Male 3×Tg-AD and WT mice were used to evaluate in vivo structural and/or functional changes in the retina and visual cortex at 4, 8, 12 and 16 months of age. The animals were maintained at 22 ± 1 °C, 68% relative humidity, in a 12 h light/12 h dark cycle, with access to water and food ad libitum. All procedures involving animals were approved by the Animal Welfare Committee of the Coimbra Institute for Clinical and Biomedical Research (iCBR), Faculty of Medicine, University of Coimbra. The animal experimentation was conducted in accordance with the European Community directive guidelines for the use of animals in laboratory (2010/63/EU), transposed into the Portuguese law in 2013 (Decreto-Lei 113/2013), and with the Association for Research in Vision and Ophthalmology (ARVO) statement for animal use.

### OCT imaging

Retinal structure was evaluated using OCT, which then allowed to perform the segmentation of retinal layers. In order to evaluate in vivo structural changes, a longitudinal study was carried out at 4, 8, 12 and 16 months of age using 3×Tg-AD and age-matched WT mice. In vivo OCT line and circular scans were acquired with Phoenix OCT2 together with Phoenix Micron IV retinal imaging microscope (Phoenix Research Laboratories, San Ramon, CA, USA). Retinal thickness was measured using InSight image segmentation software (v.1, Voxeleron LLC - Image analysis solutions, Chabot Drive, CA, USA) between the inner limiting membrane and the retinal pigment epithelium. For each eye, one OCT scan was performed around the optic nerve head and six scans were performed above and below the optic nerve head (Additional file 1: Figure S1A). Subsequently, measurements of ganglion cell layer (GCL) plus the inner plexiform layer (IPL), inner nuclear layer (INL) plus outer plexiform layer (OPL), outer nuclear layer (ONL), inner segments (IS) plus outer segments (OS) and total retina thickness were obtained both for 3×Tg-AD and WT animals (Additional file 1: Figure S1B). Circular and line scan thickness values for each animal are presented as the average value of both eyes. To perform OCT, animals were anesthetized using a combination of ketamine+xylazine 80 mg/kg + 5 mg/kg (Imalgene 1000, Merial, Lyon, France, and Rompum®, Bayer, Leverkusen, Germany, respectively) administered with one intraperitoneal injection. Pupils were dilated using topical tropicamide (1%, Tropicil®, Laboratório Edol, Carnaxide, Portugal). The cornea was anesthetized with topical anesthetic eye drops (4 mg/ml oxybuprocaine hydrochloride, Anestocil®, Laboratório Edol, Carnaxide, Portugal). The cornea was kept hydrated and optically cleared using hydroxypropyl methylcellulose (Methocel™ 2%, Dávi II Farmacêutica S.A., Barcarena, Portugal), during the whole procedure.

### Electroretinography

Retinal physiology was evaluated using flash and pattern electroretinography (fERG and PERG, respectively), in which electrical response of retinal cells is measured. PERG allowed for the assessment of the function of retinal ganglion cells (RGCs). Assays were performed at 4, 8, 12, and 16 months of age in 3×Tg-AD and age-matched WT mice.

#### Flash electroretinography

After 12 h overnight dark adaptation, mice were anesthetized with an intraperitoneal injection of ketamine (80 mg/kg) and xylazine (5 mg/kg) in 0.1 ml saline solution, under dim red light illumination. Pupil was dilated using topical phenylephrine (100 mg/ml phenylephrine hydrochloride, Davinefrina, Dávi II Farmacêutica S.A.), and the cornea was locally anesthetized and kept hydrated, as described in the previous section. The body temperature was maintained with a heating pad set to 37 °C. ERG recordings were acquired using a RETIport System (Roland Consult Electrophysiological Diagnostic Systems, Brandenburg, Germany), based on a protocol described previously (Rosolen et al., 2005). The gold ring electrode (gold wire 0.25 mm, 2.5 mm diameter, Roland Consult) was placed at the corneal surface, the reference electrode was placed subcutaneously at the head, and the ground electrode was placed also subcutaneously in the base of the tail. Light stimulation was performed using a Ganzfeld stimulator (Roland Consult). Series of white flashes of seven different light intensities (0.0095 to 9.49 cd.s/m^2^) were delivered three times at 0.1 and 1.3 Hz for the acquisition of the scotopic and photopic luminance responses, respectively. Photopic adaptation to a white background (25 cd/m^2^) was carried out during 16 min, and light flashes with intensity of 9.49 cd.s/m^2^ were applied three times at 1.3 Hz, at the onset of light adaptation and at 2, 4, 8, and 16 min of light adaptation. For the photopic flicker response, under a white background light (25 cd.s/m^2^), white bright flashes (3.00 and 9.49 cd.s/m^2^) were delivered ten times at 6.3 Hz. ERGs were recorded with a bandwidth of 1–300 Hz at a sampling rate of 3.4 kHz (0.8 kHz for flicker test). Measurement of negative *a*-wave, positive *b*-wave and individual oscillatory potentials (OPs) amplitude and time to peak values, were made using RETIport software (Roland Consult). Scotopic *a*-wave and *b*-wave amplitudes were obtained for seven light intensities from 0.0095 to 9.49 cd.s/m^2^. Four OPs were extracted for six light intensities between 0.030 and 9.49 cd.s/m^2^. During photopic adaptation, the *b*-wave amplitude was measured. *b*-wave amplitude photopic luminance responses were measured for 3.00 and 9.49 cd.s/m^2^. OFF-line digital filter was applied on the *b*-wave (high-frequency cut-off of 50 Hz) and oscillatory potentials (low-frequency cut-off of 60 Hz for scotopic ERGs and 55 Hz for photopic ERGs). Flicker responses were evaluated by determining the amplitude and phase of the base wave (6.33 Hz), first (12.7 Hz), and second (19 Hz) harmonics with the fast Fourier transform.

#### Pattern electroretinography

Animals were prepared as described in the previous section. Light-adapted PERG recordings were acquired using the RETIport System (Roland Consult). Electrodes were placed in the animal, as described above. Stimuli were dark and light gratings displayed on a monitor whose center was aligned with the projection of the pupil and presented from a short distance (typically, 20 cm) to stimulate a large retinal area. Stimuli were displayed for 200 sweeps of contrast reversals with 1 Hz temporal frequency, contrast between 95 and 100%. After PERG recording of the right eye, a new electrode was placed on the left eye and the same procedure was followed. RGC activity was evaluated by measuring an initial corneal positive response, referred to as P50, followed by a corneal negative response, referred to as N95. An evaluation of the amplitude of P50/P1 and N95/N2, the time to peak of the major positive and negative deflection was performed. Signals were band pass filtered (1–40 Hz) and artifact rejection was set at 100 μV peak to peak with a sample frequency of 1.136 kHz.

### Magnetic resonance imaging (MRI)

Brain structure was evaluated using magnetic resonance imaging (MRI). Data acquisition was performed at 4, 8, 12, and 16 months of age in 3×Tg-AD and age-matched WT mice.

#### Data acquisition

MRI experiments were performed in a BioSpec 9.4 T scanner with a standard cross coil setup using a volume coil for excitation (with 86/112 mm of inner/outer diameter, respectively) and quadrature mouse surface coil for signal detection (Bruker Biospin, Ettlingen, Germany). Animals were kept anesthetized with isoflurane (1.5%) with 100% O_2_ and body temperature and respiration monitoring (SA Instruments SA, Stony Brook, NY, USA). T2-weighted images were acquired in coronal planes using a Rapid Acquisition with Relaxation Enhancement (RARE) sequence with the following parameters: TR = 3800 ms; TE = 33 ms; 10 averages; pixel size of 0.078 mm × 0.078 mm and slice thickness of 0.5 mm without spacing between slices (total head volume: 256 pixels × 256 pixels × 34 slices).

#### Data analysis

Voxel-based morphometry (VBM) was performed using the statistical parametric mapping software (SPM, Welcome Department of Cognitive Neurology, London, UK), the SPMMouse toolbox [55], and a homemade script involving the following steps: (a) T2-weighted images were corrected for the magnetic field inhomogeneity generated by the surface coil and this was done using intensity curves from T2-weighted images obtained for an homogeneous phantom and acquired with the same coil and system configuration; (b) the rigid body was aligned by registering (affine transformation) the images to the template space; (c) tissue segmentation was carried out by means of the gray matter (GM), white matter (WM), and cerebrospinal fluid (CSF) tissue probability maps as provided in the toolbox; (d) GM images were non-linearly normalized to the template space and were modulated to correct for volume changes that might have occurred during normalization; (e) a binary mask, corresponding to the right visual cortex, was drawn in the template space and placed in each aligned GM image; (f) finally, the GM volume was quantified by multiplying the GM volume in each voxel by the number of voxels. All steps were done using the default settings of the toolbox.

### Statistical analysis

Statistical analysis was performed using GraphPad Prism Version 6 (GraphPad Software, San Diego, CA, USA) and SPSS 22.0 (SPSS Inc., Chicago, IL, USA). The normality of the data was assessed with D’Agostino and Pearson omnibus normality tests. Differences in thickness of retinal layers and in retinal function between groups, at each time point (4, 8, 12, and 16 months) were assessed using an analysis of variance (ANOVA) repeated-measures (mixed-effects), where time was assumed as “within-subject” factor, and the experimental group (i.e., 3×Tg-AD and WT mice) was assumed as “between-subject” factor, followed by a post hoc *t*-test. Differences in GM volumes of the visual cortex along time and between groups were assessed using an ANOVA repeated measures (mixed-effects) followed by a Bonferroni post hoc *t*-test. Results were presented as mean ± SEM. Statistical significance was considered at *p < 0.05, **p < 0.01, and ***p < 0.001.

## Results

### Reduction of retinal thickness of 3×Tg-AD mice

**Fig. 1:**
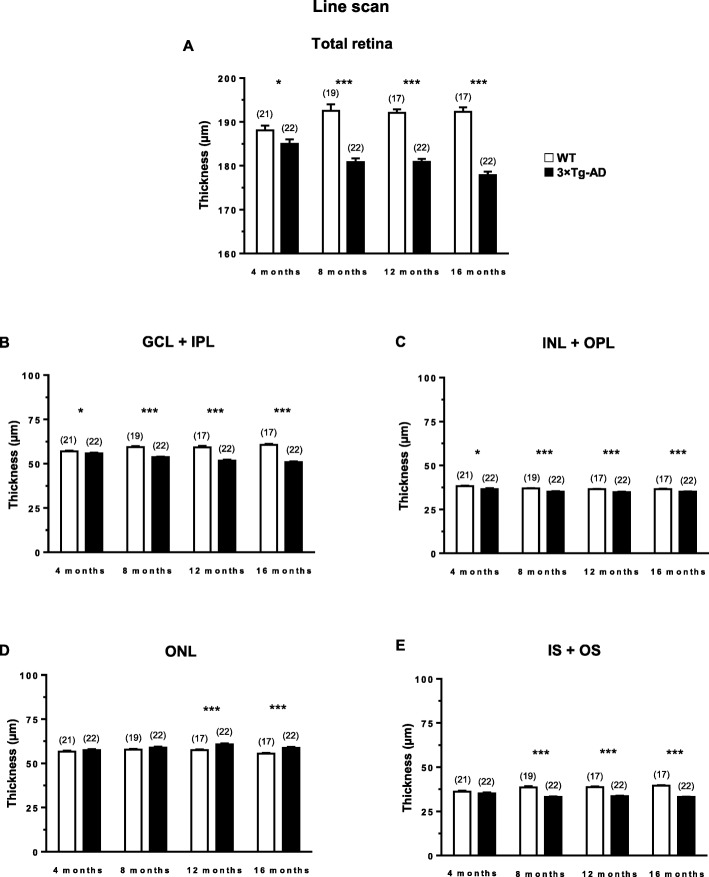
Thickness of different retinal layers in WT (white bars) and 3×Tg-AD (black bars) mice at 4, 8, 12, and 16 months of age, based on in vivo OCT line scans. The thickness of retinal layers was measured using the InSight software. **a** Total retina, **b** GCL+IPL, **c** INL+OPL, **d** ONL, **e** IS+OS. The results are presented as mean ± SEM. *p < 0.05, ***p < 0.001, according to Student’s *t* test. *n*_WT_: at 4 months = 21, at 8 months = 19, at 12 months = 17, at 16 months = 17; *n*_3×Tg-AD_: at 4 months = 22, at 8 months = 22, at 12 months = 22, at 16 months = 22

We evaluated, in vivo, whether the retinal structure of 3×Tg-AD mice was affected, using OCT (line and circle scans; Additional file 1: Figure S1), followed by the segmentation of retinal layers. ANOVA revealed that the total retina thickness was significantly decreased in 3×Tg-AD mice compared to that in WT mice (line scans, *F* (1, 33) = 100.2, *p* < 0.001; circle scans, *F* (1, 31) = 106.0, *p* < 0.001) (Fig. 1a, Additional file 1: Figure S3A). However, no time effect was observed (line scans, *F* (3, 99) = 1.3, *p* = 0.270) (Fig. 1a). Furthermore, throughout the study, we observed a significant thinning of GCL+IPL (*F* (2.6, 85.7) = 3.4, *p* < 0.05) and INL+OPL (*F* (2.2, 72.5) = 19.0, *p* < 0.001), whereas no time effect was observed on the IS+OS layer thickness (*F* (2.5, 82.8) = 0.73, *p* = 0.510). The thickness of each retinal layer was consistently significantly lower in 3×Tg-AD mice compared to WT mice (GCL+IPL, *F* (1, 33) = 121.8, *p* < 0.001; INL+OPL, *F* (1, 33) = 22.7, *p* < 0.001; IS+OS layer, *F* (1, 33) = 105.1, *p* < 0.001) (Fig. 1). Particularly, at 16 months, the total retinal thickness of WT mice, 192.3 μm, decreased to 177.9 μm in 3×Tg-AD mice (*p* < 0.001, Fig. 1a), and the thickness of GGL+IPL decreased from 60.7 μm in WT mice to 51.0 μm in 3×Tg-AD mice (*p* < 0.001, Fig. 1b), whereas the IS+OS layer thickness was 39.5 μm and 33.1 μm in WT and 3×Tg-AD mice, respectively (*p* < 0.001, Fig. 1e). However, conversely to the results obtained for the layers described above, a significant increase of ONL thickness (*F* (2.6, 85.1) = 8.6, *p* < 0.001) was observed, during the study. Also the thickness of ONL of 3×Tg-AD mice was significantly higher (*F* (1, 33) = 8.7, *p* < 0.01) in comparison with WT mice (Fig. 1d). At 16 months, the ONL thickness was 58.8 μm and 55.5 μm in 3×Tg-AD and WT mice, respectively (*p* < 0.001). Overall, we observed a thinning of total retina, GCL+IPL, INL+OPL, and IS+OS of 3×Tg-AD mice. Conversely, ONL was found to be thicker in this AD animal model.

### Alterations in the retinal physiological scotopic responses of 3×Tg-AD animals

We used fERG to explore the functional integrity of specific retinal cell types in both WT and 3×Tg-AD mice. Regarding the scotopic response, the retina is stimulated with low-intensity light flashes to induce rod activation. The function of photoreceptors and downstream retinal cells can then be examined. Photoreceptors (mainly rods) are the cells contributing to *a*-wave, whereas the *b*-wave is widely believed to reflect mainly the activation of bipolar cells. OPs are thought to reflect cell activity in the inner retina, mainly amacrine cells [56].

**Fig. 2:**
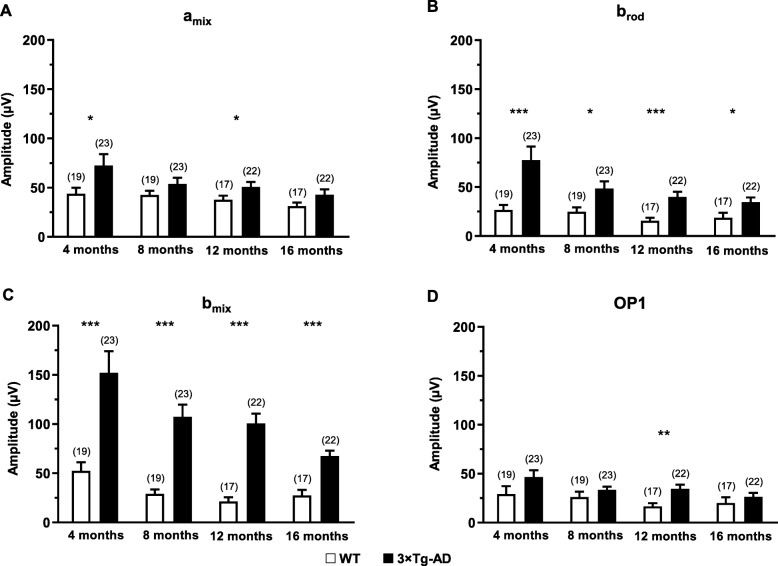
Retinal function in WT and 3×Tg-AD mice at 4, 8, 12, and 16 months of age assessed by fERG, under scotopic conditions. Main component values obtained in response to 0.0095 cd.s/m^2^ light stimulus (b_rod_) or 9.49 cd.s/m^2^ light stimulus (*a*_mix_, *b*_mix_, and OP1) in WT (white bars) and 3×Tg-AD (black bars): **a** *a*_mix_ amplitude, **b** *b*_rod_ amplitude, **c** *b*_mix_ amplitude, **d** OP1 amplitude. The results are presented as mean ± SEM. *p < 0.05, **p < 0.01, and ***p < 0.001 according to Student’s *t* test. *n*_WT_: at 4 months = 19, at 8 months = 19, at 12 months = 17, at 16 months = 17; *n*_3×Tg-AD_: at 4 months = 23, at 8 months = 23, at 12 months = 22, at 16 months = 22

**Fig. 3:**
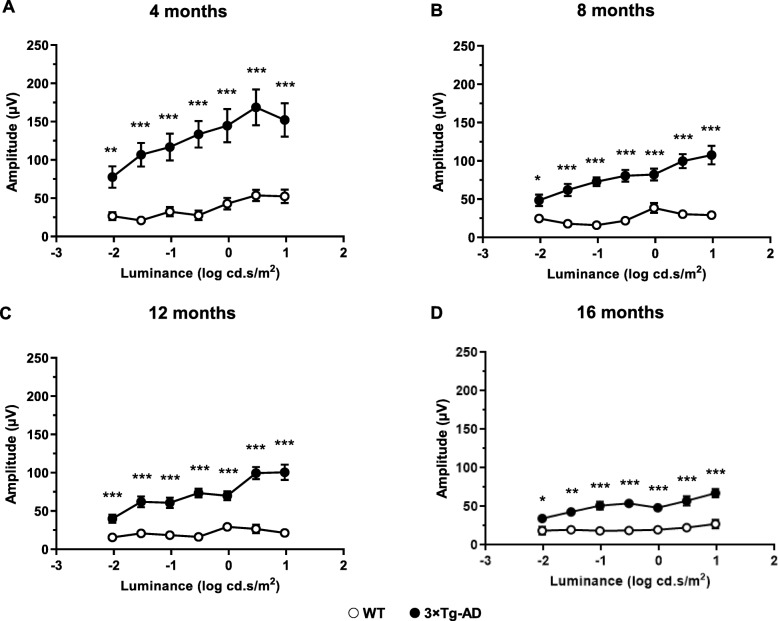
Scotopic *b*-wave amplitude in WT (white circles) and 3**×**Tg-AD (black circles) mice, at **a** 4, **b** 8, **c** 12, and **d** 16 months of age. Graphs present the amplitude of the *b*-wave at the indicated luminance conditions. The results are presented as mean ± SEM. *p < 0.05, **p < 0.01, ***p < 0.001, according to Student’s *t* test. *n*_WT_: at 4 months = 19, at 8 months = 19, at 12 months = 17, at 16 months = 17; *n*_3**×**Tg-AD_: at 4 months = 23, at 8 months = 23, at 12 months = 22, at 16 months = 22

The results obtained from the analysis of these electroretinograms showed that, overall, 3×Tg-AD mice had significantly higher scotopic *a*-wave amplitude than age-matched WT mice (*F* (1, 30) = 10.6, *p* < 0.01), particularly, at 4 and 12 months (*p* < 0.05, Fig. 2a). ANOVA further determined significant differences in scotopic *a*-wave amplitude during the study (*F* (2.0, 62.8) = 4.0, *p* < 0.05). Our results also showed that 3×Tg-AD mice present lower time to peak values in comparison with age-matched WT animals, mainly at higher luminance (*F* (1, 28) = 130.2, *p* < 0.001, Additional file 1: Figure S4). Therefore, we observed a faster response in the *a*-wave of 3×Tg-AD mice. The amplitude of scotopic *b*-wave resulting from rod activation (*b*_rod_, *F* (1, 30) = 33.2, *p* < 0.001) and from rod and cone activation (*b*_mix_, *F* (1, 30) = 52.6, *p* < 0.001) of 3×Tg-AD mice was significantly higher than age-matched WT mice, regardless of the time point (Fig. 2b, c, respectively). Actually, clear differences between 3×Tg-AD and age-matched WT mice were observed at 4, 8, 12, and 16 months of age, regardless of the luminance (*F* (1, 28) = 35.5, *p* < 0.001, Fig. 3). Moreover, we observed a reduction of the *b*-wave amplitude difference between 3×Tg-AD and WT mice, from 4 to 16 months of age (*F* (3, 84) = 8.6, *p* < 0.001, Additional file 1: Figure S5). We further found a significant reduction of *b*_rod_ (*F* (1.9, 55.5) = 4.5, *p* < 0.05) and *b*_mix_ (*F* (1.9, 56.3) = 9.8, *p* < 0.001) amplitude, along time.

The OP1 amplitude values of 3×Tg-AD mice were higher than those of age-matched WT mice, at all time points (*F* (1, 30) = 7.3, *p* < 0.05). However, statistically significant differences (*p* < 0.01) were observed only at 12 months (Fig. 2d). A significant decrease throughout the study was further observed (*F* (3, 90) = 3.1, *p* < 0.05). OP1 time to peak values were also affected. We observed that 3×Tg-AD mice presented faster OP responses (*F* (1, 29) = 28.4, *p* < 0.001, Additional file 1: Figure S6). During the study, no significant differences were found (*F* (2.6, 74.8) = 2.5, *p* = 0.074). No statistically significant interaction time and experimental group was observed in scotopic response. In summary, under scotopic conditions, we found several alterations in *a*-wave, *b*-wave, and OPs of 3×Tg-AD, regarding amplitude and time to peak, denoting clear physiological alterations in the retina of this AD animal model.

### Increased amplitude of photopic responses of 3×Tg-AD mice

**Fig. 4:**
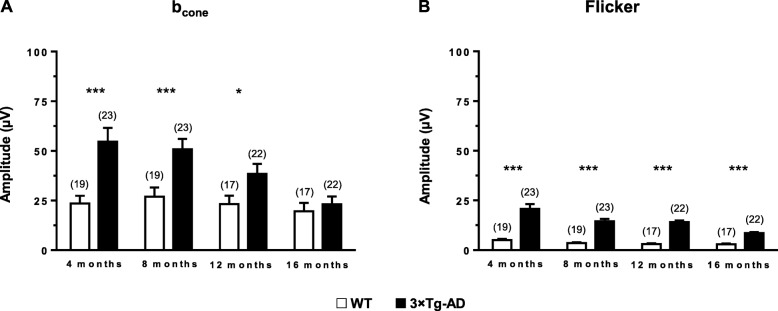
Retinal function in WT and 3×Tg-AD mice at 4, 8, 12, and 16 months of age, assessed by fERG, under photopic conditions. Main component values obtained in response to 9.49 cd.s/m^2^ light stimulus in WT (white bars) and 3×Tg-AD (black bars): **a** *b*_cone_ amplitude, **b** base wave amplitude. The results are presented as mean ± SEM. **p < 0.01, ***p < 0.001 according to Student’s *t* test. *n*_WT_: at 4 months = 19, at 8 months = 19, at 12 months = 17, at 16 months = 17; *n*_3×Tg-AD_: at 4 months = 23, at 8 months = 23, at 12 months = 22, at 16 months = 22

Once fully light-adapted, the retina was stimulated with high-intensity light flashes. Thus, during the photopic response, the function of cone photoreceptors is measured, since the rod response is suppressed [56]. The *b*-wave amplitude of 3×Tg-AD mice was significantly higher (*F* (1, 30) = 49.5, *p* < 0.001) than WT mice. However, no statistically significant differences were observed at 16 months (Fig. 4a). Throughout the study, we observed a statistically significant decrease in the *b*-wave amplitude (*F* (3, 90) = 4.0, *p* < 0.01), particularly between 4 (*p* < 0.05) and 8 (*p* < 0.01) months vs 16 months. The interaction time and experimental group was also found to be statistically significant (*F* (3, 90) = 2.8, *p* < 0.05). Accordingly, similarly to scotopic conditions, retinal photopic responses were altered in the retina of 3×Tg-AD mice.

### Increased photopic flicker harmonic amplitude of 3×Tg-AD mice

**Fig. 5:**
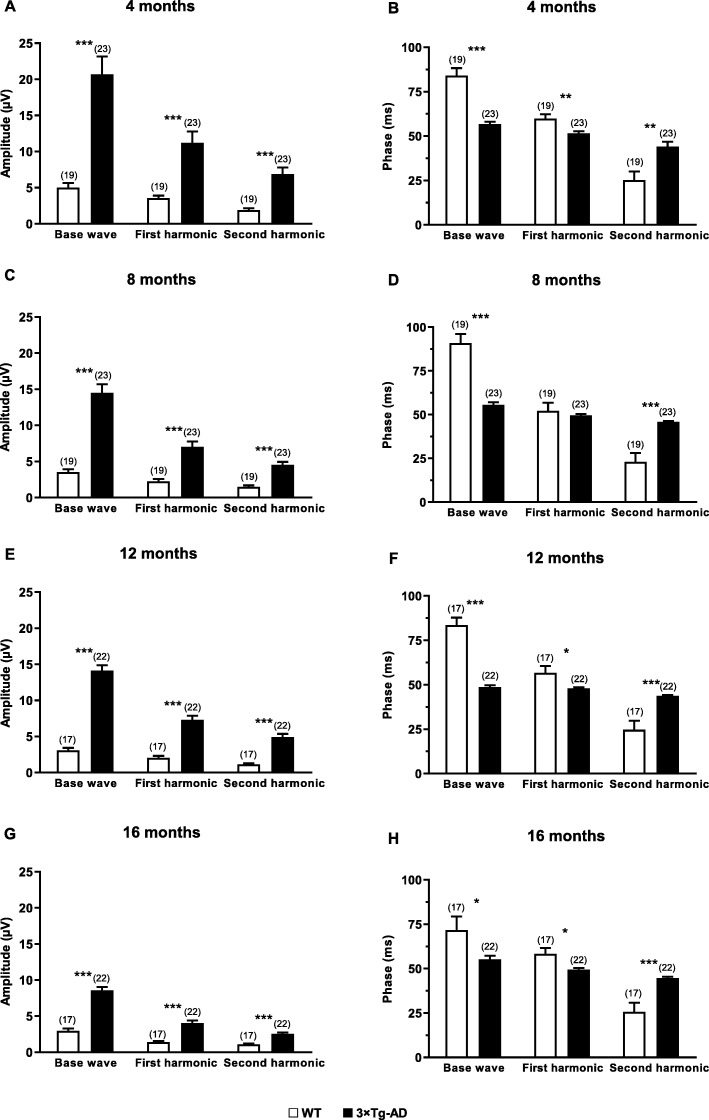
Photopic flicker retinal response in WT (white bars) and 3×Tg-AD (black bars) mice at **a**, **b** 4, **c**, **d** 8, **e**, **f** 12, and **g**, **h** 16 months of age. The responses were recorded in response to 9.49 cd.s/m^2^ light stimulus. The amplitude and phase of the signal were evaluated after fast Fourier transform. The results are presented as mean ± SEM. **p < 0.01, ***p < 0.001, according to Student’s *t* test. *n*_WT_: at 4 months = 19, at 8 months = 19, at 12 months = 17, at 16 months = 17; *n*_3×Tg-AD_: at 4 months = 23, at 8 months = 23, at 12 months = 22, at 16 months = 22

As in the photopic response, in the photopic flicker response the electrical activity is mainly cone-driven. The base wave (*F* (1, 30) = 160.0, *p* < 0.001), first (*F* (1, 30) = 57.0, *p* < 0.001), and second (*F* (1, 30) = 83.6, *p* < 0.001) harmonic amplitudes of 3×Tg-AD mice was significantly higher compared to age-matched WT mice, at all time points (Fig. 4b and Fig. 5a, c, e, g). We also found an overall significant decrease of base wave amplitude values, throughout the different time points (*F* (1.6, 49.1) = 14.8, *p* < 0.001, Additional file 1: Figure S7A). The interaction time and experimental group was also found to be statistically significant (*F* (1.6, 49.1) = 6.0, *p* < 0.01). The first harmonic amplitude values also decreased over time (*F* (1.5, 45.5) = 14.6, *p* < 0.001, Additional file 1: Figure S7C). The interaction time and experimental group was statistically significant as well (*F* (1.5, 45.5) = 4.0, *p* < 0.05). The second harmonic amplitude values significantly decrease overtime (*F* (1.5, 46.1) = 11.8, *p* < 0.001, Additional file 1: Figure S7E). Regarding the interaction time and experimental group, statistically significant differences were found (*F* (1.5, 46.1) = 6.3, *p* < 0.01).

Moreover, 3×Tg-AD animals tend to follow the input base wave frequency (6.33 Hz) more effectively than WT mice leading to a difference in phase responses (*F* (1, 30) = 100.8, *p* < 0.001) (Fig. 5b, d, f, h and Additional file 1: Figure S7B). In the first harmonic phase, a similar result was obtained, meaning that 3×Tg-AD mice still had a faster response at first harmonic frequency (12.7 Hz) than WT mice (*F* (1, 30) = 21.9, *p* < 0.001) (Fig. 5 b, d, f, h and Additional file 1: Figure S7D). Conversely, WT mice were found to have a faster response at second harmonic frequency (*F* (1, 30) = 80.8, *p* < 0.001) (19 Hz, Fig. 5b, d, f, h and Additional file 1, Figure S7F). No statistically significant time effect was observed in the flicker responses, nor any interaction time and experimental group. Overall, we observed higher amplitude and lower phase values in the photopic flicker response of 3×Tg-AD.

### Similar PERG responses of 3×Tg-AD and WT mice

**Fig. 6:**
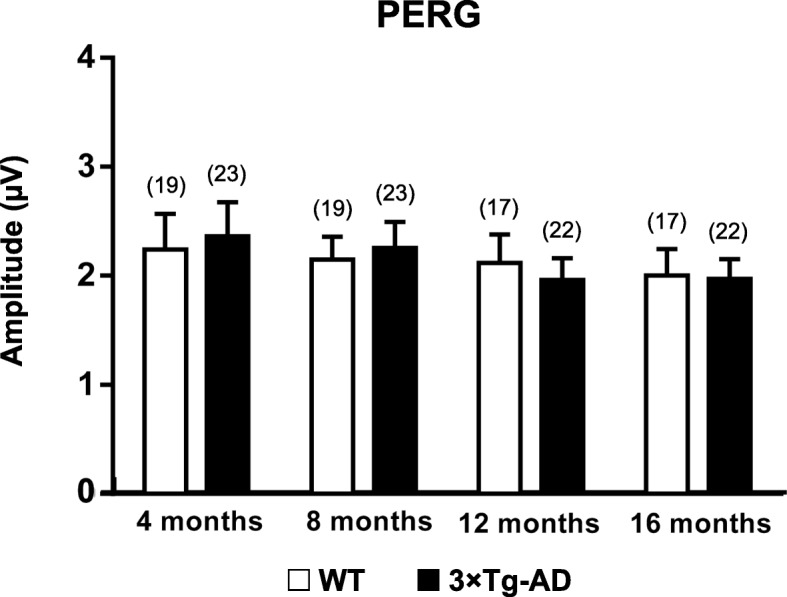
RGC function in WT and 3×Tg-AD mice at 4, 8, 12, and 16 months of age, assessed by PERG. PERG amplitude obtained in WT (white bars) and 3×Tg-AD (black bars). The results are presented as mean ± SEM. No statistically significant differences were obtained, according to Student’s *t* test. *n*_WT_: at 4 months = 19, at 8 months = 19, at 12 months = 17, at 16 months = 17; *n*_3×Tg-AD_: at 4 months = 23, at 8 months = 23, at 12 months = 22, at 16 months = 22

Electrophysiological assessment of RGC function was made using PERG. Regarding amplitude, no statistically significant differences were observed between 3×Tg-AD and age-matched WT mice (Fig. 6). In particular, no statistically significant differences were found between both groups in P1 and N2 implicit time and amplitude, at all time points (data not shown).

### Volume reduction of visual cortex gray matter of 3×Tg-AD mice

**Fig. 7:**
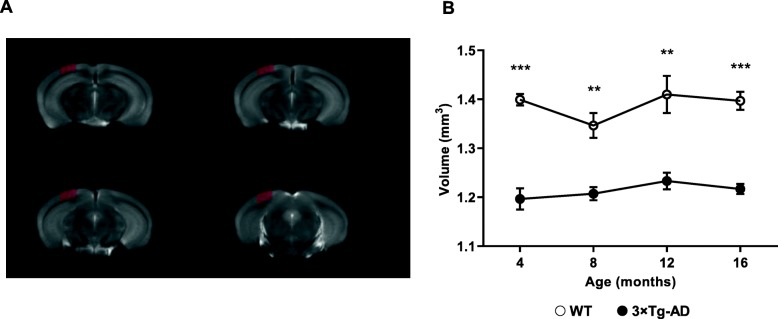
Gray matter volume analysis in the visual cortex. **a** Region of interest used in the assessment of the visual cortex volume overlaid on a mean T2-weighted image. **b** GM volume in the visual cortex of WT (white circles) and 3×Tg-AD (black circles) mice measured by VBM analysis. Results are presented as mean ± SEM. The significance of the alterations in GM volumes of the visual cortex along time and between groups was assessed by an ANOVA repeated measures (mixed-effects) followed by a Bonferroni post hoc test. **p < 0.01 and ***p < 0.001. *n*_WT_ = 6, *n*_3×Tg-AD_ = 7

We acquired whole-brain anatomical MRI data to study potential alterations in the GM volume of 3×Tg-AD mice relatively to age-matched WT mice, at different time points (4, 8, 12, and 16 months of age). A whole-brain analysis revealed a significant age-dependent reduction of the GM volume between both groups (*F* (1, 8) = 71.0, *p* < 0.001). Furthermore, an analysis focused on the visual cortex, as a region-of-interest (ROI) (Fig. 7a), also showed that the GM volume of the 3×Tg-AD mice was significantly reduced compared to the age-matched WT mice (*F* (1, 8) = 105.0, *p* < 0.001), at all time points (Fig. 7b). VBM analysis showed a significant decrease in 3×Tg-AD animals in comparison to age-matched WT mice, at all time points (Fig. 7b).

### Retinal thickness changes and physiological responses are correlated with visual cortex gray matter volume

In order to further elucidate the correlation between the alterations in the retina and visual cortex, we performed a correlation analysis between the retinal thickness and photopic flicker response with the GM volume in the visual cortex. The Spearman correlation test showed a positive correlation between total retinal thickness and GM volume (retina line scan: *r*_S_ = 0.657, *p* < 0.001; retina circle scan: *r*_S_ = 0.728, *p* < 0.001; Additional file 1: Figure S8A). Additionally, we performed the same analysis to evaluate the correlation between photopic flicker response amplitude and GM volume in the visual cortex. We found a significant negative correlation between photopic flicker base amplitude and GM volume in the visual cortex (*r*_S_ = − 0.790, *p* < 0.001; Additional file 1: Figure S8B). Moreover, the correlation of retinal thickness, namely for both line and circle scans in the GCL+IPL and the photopic flicker response amplitude was found to be negative, at all time points (Table 1).

**Table 1:** Correlations of photopic flicker (base) amplitude with retina and retinal cell layers thickness

	4 months	8 months	12 months	16 months
Total retina (line scan)	*r*_S_ = − 0.2401 ns	*r*_S_ = − 0.6554***	*r*_S_ = − 0.6920***	*r*_S_ = − 0.6822***
Total retina (circle scan)	*r*_S_ = − 0.5258**	*r*_S_ = − 0.6276***	*r*_S_ = − 0.7620***	*r*_S_ = − 0.7157***
GCL+IPL (line scan)	*r*_S_ = − 0.3833*	*r*_S_ = − 0.6554***	*r*_S_ = − 0.6317***	*r*_S_ = − 0.6763***
GCL+IPL (circle scan)	*r*_S_ = − 0.4070*	*r*_S_ = − 0.6970***	*r*_S_ = − 0.6937***	*r*_S_ = − 0.7108***
INL+OPL (line scan)	*r*_S_ = − 0.2372 ns	*r*_S_ = − 0.5290**	*r*_S_ = − 0.5396**	*r*_S_ = − 0.3801*
INL+OPL (circle scan)	*r*_S_ = − 0.0345 ns	*r*_S_ = − 0.3232 ns	*r*_S_ = − 0.5885***	*r*_S_ = − 0.4265*
ONL (line scan)	*r*_S_ = − 0.0108 ns	*r*_S_ = − 0.0792 ns	*r*_S_ = 0.5521**	*r*_S_ = 0.4663**
ONL (circle scan)	*r*_S_ = 0.0296 ns	*r*_S_ = − 0.0016 ns	*r*_S_ = 0.3765*	*r*_S_ = 0.5493**
IS+OS (line scan)	*r*_S_ = − 0.1039 ns	*r*_S_ = − 0.7188***	*r*_S_ = − 0.7614***	*r*_S_ = − 0.6525***
IS+OS (circle scan)	*r*_S_ = − 0.3707*	*r*_S_ = − 0.6885***	*r*_S_ = − 0.8194***	*r*_S_ = − 0.7771***

(*n*_circle scan_ = 30, *n*_line scan_ = 32). *r*_S_ quantifies Spearman correlation. *ns* not significant, *p < 0.05, **p < 0.01, and ***p < 0.00

Results are summarized in Table 2.

**Table 2:** Summary of the main retinal (structural and functional) and brain (visual cortex) changes in 3×Tg-AD mice

Retinal structure (layer thickness)							
Total retina	GCL+IPL		INL+OPL		ONL	IS+OS	
↓	↓		↓		↑	↓	
Retinal function							
ERG							
Scotopic				Photopic	Flicker		
*a*_mix_	*b*_rod_	*b*_mix_	OP1	*b*_cone_	Base wave	First harmonic	Second harmonic
↑ Amplitude	↑ Amplitude	↑ Amplitude	↑ Amplitude	↑ Amplitude	↑ Amplitude	↑ Amplitude	↑ Amplitude
↓ Time to peak			↓ Time to peak		↓ Phase	↓ Phase	↑ Phase
PERG							
No changes							
Brain (visual cortex)							
↓ Gray matter volume							

## Discussion

Recently, in a longitudinal study (4, 8, 12, and 16 months of age) using a mouse model of AD, 3×Tg-AD, we reported that this model presents, since the early time points, (i) recognition memory impairment, (ii) increased levels of Aβ and hyperphosphorylated tau in the hippocampus, (iii) decreased hippocampal volume, and (iv) loss of taurine, which is an important endogenous neuroprotector. We showed that hippocampal volumetry and neurospectroscopy can be early valuable biomarkers, which parallel neurobehavioral deficits, thus providing an in vivo multimodal validation of the 3×Tg-AD mouse as an early disease model [52].

In the present study, we also used 3×Tg-AD mice to assess if the retina can be a window or a mirror of the brain. Again, we performed a longitudinal multimodal in vivo assessment at four different time points (4, 8, 12, and 16 months of age), both in the retina and brain. Here, correlations between changes in the retina and brain were performed, based on different physiological and structural approaches in association with MRI. This study also represents not only a completely new approach in animal models to tackle the potential links between retinal and brain changes in the context of AD pathology, but also gives new clues about the pathology and confirms some findings that have been already observed in humans. In the present study, only male mice were used. Recent evidences support the importance of using a balanced population of male and female subjects due to their distinct biochemical, physiological, and structural features in several organs including the brain [57–59]. It was reported that 3×Tg-AD male mice present a delayed and milder phenotype compared to female counterparts [60]. However, we recently showed that 3×Tg-AD male mice present pathological and neurobehavioral features of AD, with early-onset recognition memory loss and reduction of hippocampal volume [52]. Other studies also demonstrated that male mice present AD features [61–63]. Therefore, 3×Tg-AD male mice remain a valuable experimental model. Concerning the animal model, we further add that mice here used were dark eyes. Eventual differences in mice with dark eyes vs albino mice may be interesting to address in future studies.

Several studies in AD patients, in which retinal thickness was evaluated by OCT, have reported a thinning in the retina [21, 22, 64, 65]. Here, we replicated this finding in an animal model, suggesting that OCT may be useful for an early diagnosis of AD and also for the evaluation of the response to treatment in such preclinical models. Therefore, our work does corroborate the notion that AD animal models can be used to assess retinal changes and the impact of neurodegeneration [5]. From the best of our knowledge, the present study described, for the first time, the retinal structural and functional changes in 3×Tg-AD animal model. At 4 months of age, we observed the thinning of GCL+IPL and of total retina both for circle and line scans, in 3×Tg-AD mice. We also detected the thinning of INL+OPL in OCT line scan acquisitions. These observations suggest that the first signs of retinal impairment in AD start at the innermost layers. With this observation, we cannot discard the possibility that transsynaptic degeneration, from the brain to the retina, may occur. Another possibility is that some changes start occurring in the retina, even before or at least at the same time they start occurring in the brain, although this could not be ascertained. From 8 months onwards, we observed the thinning of total retina, GCL+IPL, INL+OPL, and IS+OS of 3×Tg-AD mice. The thinning observed in the inner retina, earlier, at 4 months, seemed to spread to almost every analyzed retinal layer. This might be due to secondary degeneration, a phenomenon in which synapses and neurons in the neighborhood of originally affected neurons are also affected. The retinal thinning here observed, even at early time points, is an important finding that corroborates previous results obtained in humans, in which OCT results clearly show that there is retinal thinning in AD patients [10, 23, 65]. Despite the generalized thinning of several layers of the retina, there was also an increase in the thickness of the ONL of 3×Tg-AD mice compared to age-matched WT mice. The thickening of ONL is not easy to explain, but we cannot exclude that it might have a developmental cause.

The aim of this research work was to perform a longitudinal in vivo study, using the same animals for the different experimental techniques utilized in each time point. We did not aim to go deeper in the structural changes of the retina and the cortex. We believe that a longitudinal in vivo study has much more relevance for the clinical setting. Moreover, OCT is the technique widely used in clinical evaluation to assess the retina and, in particular, retinal layer thickness. In the context of our study, having very small changes in retinal thickness, histological examination would not definitely be a good approach. Actually, it would be unlikely to detect significant changes using histology. Histological analysis also requires the use of different animals in each time point, which would not be feasible in a longitudinal study. Moreover, it would be impossible to obtain slices exactly in the same region of the retina whereas, by OCT, we were able to analyze the same region of the retina. Also, tissue collection and preparation for histological analysis affects retinal structure, and therefore, the thickness measured would not be as reliable. The same applies to the brain. For instance, we evaluated the volume of the visual cortex in vivo, by MRI, and this is much more reliable than calculating the volume based on histological analysis of the brain.

Regarding retinal electrophysiology, scotopic and photopic *b*-wave amplitudes of 3×Tg-AD and WT mice were significantly different at various time points. We observed alterations in *b*-wave amplitude of 3×Tg-AD mice at 4, 8, 12, and 16 months of age, which suggests the existence of physiological modifications in bipolar cells. The increased amplitude values observed in fERG responses, originated from the outer retina, are consistent with the increased ONL thickness that we found. Moreover, the scotopic time to peak in the *a*-wave and OP1 was shorter in 3×Tg-AD mice. Abnormal scotopic *b*-wave implicit time and decreased *a*-wave amplitude have been also reported in 3×Tg-AD mice [41]. Furthermore, higher scotopic and photopic *b*-wave amplitudes were previously observed in a mouse model of Down syndrome, in which specific changes in layer thickness were also found [66]. In fact, Aβ deposition has been detected both in AD and Down syndrome patients [67]. In Down syndrome patients, the neurodegenerative processes associated with AD are also expressed. Moreover, APP gene is located at chromosome 21, and Down syndrome patients have an extra copy of this chromosome, thus leading to the development of early onset dementia with AD characteristics [68, 69]. This may entail that there is a common phenotype in the retina between AD and Down syndrome patients.

Early differences in the ERG response between 3×Tg-AD and WT were observed at the photoreceptor level. In this study, WT animals were chosen according to the genetic background from which 3×Tg-AD mice were generated. It seems that the introduction of human genes that have been associated with AD had an opposite effect to what was expected, if one assumes that the effects of this genetic manipulation impact only on neurodegeneration. However, if developmental effects are also present, as suggested by the increased thickness of ONL, then this might explain increased fERG responses in 3×Tg-AD mice. In general, we observed that fERG signal amplitude, which mainly originates from the outer retina, was higher in 3×Tg-AD mice than in WT mice. However, regarding the inner retinal responses from RGC evaluated by PERG, we did not observe any statistically significant difference between both groups. In previous studies in humans, results obtained with PERG recordings are contradictory. Actually, some studies show abnormal pattern electroretinograms and visual evoked potentials in AD patients [70–73], whereas others report normal retinal function [27, 74, 75]. In PERG recordings, P1 is driven by ON pathway while N2 is driven by OFF pathway [76]. We did not observe statistically significant differences in P1 and N2 parameters between 3×Tg-AD and WT mice meaning that RGC function is not significantly impaired in this AD animal model.

Since both structural and functional changes were observed in the retina, at all time points, the possible causal links (early neurodegenerative or neurodevelopmental) on structure and function remain somehow elusive. In order to better understand the retinal physiological changes in this AD animal model, it would be essential to perform either multielectrode array or patch clamp recordings, which would enable to determine more precisely the changes in retinal response properties to a given light stimulus. Visual evoked potential recordings would enable to determine if visual information transmitted from the retina to the brain is impaired.

Previous studies have been published reporting retinal molecular and cellular changes in 3×Tg-AD mice. Profound alterations in retinal tau, including abnormal accumulation, phosphorylation, and missorting were already described in 3×Tg-AD mice, at 3 months of age [77]. These pathological changes are likely to cause substantial retinal neuron dysfunction and subsequent death. The presence of Aβ plaques, tau tangles, neurodegeneration, and astrogliosis in the retinal ganglion cell layer was described in 3×Tg-AD mice, at 1–5 months of age. An anti-inflammatory phenotype of retinal microglial cells, at 1–5 months, which then evolves to a pro-inflammatory phenotype, at 12–18 months, was also described [78]. Several proteins related to oxidative stress, light-dependent processes (e.g., Sag), synaptic functions, metabolism, and energy production in retina were found to be dysregulated in 3×Tg-AD mice, at 2, 4, and 6 months of age [79]. It is therefore clear that there are several molecular and cellular alterations in the retina of 3×Tg-AD, even at early time points, which can contribute to functional alterations that we observed. However, it is difficult to identify a particular molecular or cellular alteration that is responsible for a particular electrophysiological alteration.

Our results also suggest that there is a positive correlation between retinal thickness and GM volume of the visual cortex, while ERG flicker amplitude is negatively correlated with retinal and cortical structural measurements. However, it is quite difficult to identify the biological basis for these nonlinear (Spearman) correlations and, for example, why changes occurring in the visual cortex could contribute for the thinning or thickening of the retina. In fact, there may be several causes, such as developmental impacts or other indirect effects, that could lead to these correlations. In our opinion, similar pathophysiological mechanisms may occur in the brain and retina, since both structures are part of the CNS. However, what is happening in the visual cortex does not necessarily affect, at least directly, the retina. These associations further suggest that enhanced ERG responses may actually reflect a retinal impairment, as also suggested by Laguna and colleagues [59]. Accordingly, we show that retinal structural and functional changes are associated with visual cortex alterations, thus having potential impact on early AD diagnosis.

## Conclusions

In this longitudinal in vivo study, the 3×Tg-AD mouse model allowed the identification of structural and neurophysiological alterations in the retina, as well as changes in gray matter volume in the visual cortex. These retinocortical alterations showed an early onset and remained persistent over time. Some of these changes appear to be of neurodegenerative nature, as expressed by retinal and brain thinning. Since the retina is an optically accessible part of the brain, OCT may become a feasible, noninvasive, low-cost method and an additional tool for preclinical research in AD. In addition, the 3×Tg-AD mouse model may be used as a potential tool for preclinical studies in which retinal structural and functional changes could be correlated with brain impairments detected by neuroimaging, which may be quite useful for preclinical drug trials. The results emerging from the present research assume particular importance because they enable the establishment of a model in which retinal manifestations of AD are observed at an early time point. Moreover, our results emphasize the possibility of retinal biomarker assessment in AD.

## Supplementary information

**Additional file 1: Figure S1.** OCT scan acquisition. **Figure S2.** Retinal layers visualized by OCT. **Figure S3.** Thickness of different retinal layers in WT and 3×Tg-AD mice at 4, 8, 12 and 16 months of age, based on in vivo OCT circle scans. **Figure S4.** Scotopic *a*-wave time to peak in WT and 3×Tg-AD mice, at 4, 8, 12 and 16 months of age, as response to several light stimuli. **Figure S5.** Linear regression fit to scotopic *b*-wave amplitude at 0.0095 cd.s/m^2^ and 9.49 cd.s/m^2^ in WT and 3×Tg-AD mice. **Figure S6.** OP1 time to peak values at 4, 8, 12 and 16 months of age, based on fERG analysis. **Figure S7.** Linear regression fit to base wave, first harmonic and second harmonic amplitudes and phases in WT and 3×Tg-AD mice. **Figure S8.** Spearman correlation between total retinal thickness and grey matter volume of the visual cortex, and photopic flicker response amplitude and grey matter volume of the visual cortex.

## Data Availability

All data generated or analyzed during this study are included in this published article and its additional files.

## Abbreviations

3×Tg-AD: Triple transgenic mouse model of Alzheimer’s disease
AD: Alzheimer’s disease
Aβ: Amyloid beta
CSF: Cerebrospinal fluid
ERG: Electroretinography
fERG: Flash electroretinography
GCL: Ganglion cell layer
GM: Gray matter
INL: Inner nuclear layer
IPL: Inner plexiform layer
IS: Inner segment
MRI: Magnetic resonance imaging
OCT: Optical coherence tomography
ONL: Outer nuclear layer
OP: Oscillatory potentials
OPL: Outer plexiform layer
OS: Outer segment
PERG: Pattern electroretinography
RGC: Retinal ganglion cell
RNFL: Retinal nerve fiber layer
WM: White matter
WT: Wild type
