# Supplementary material for: Retinal thinning of inner sub-layers is associated with cortical atrophy in a mouse model of Alzheimer’s disease: a longitudinal multimodal in vivo study
Source: Alzheimers Res Ther. 2019 Nov 13;11:90. doi: 10.1186/s13195-019-0542-8 (PMC6854691; doi:10.1186/s13195-019-0542-8)
Supplement: Supplementary file 1 — Additional file 1: Figure S1. OCT scan acquisition. Figure S2. Retinal layers visualized by OCT. Figure S3. Thickness of different retinal layers in WT and 3×Tg-AD mice at 4, 8, 12 and 16 months of age, based on in vivo OCT circle scans. Figure S4. Scotopic a-wave time to peak in WT and 3×Tg-AD mice, at 4, 8, 12 and 16 months of age, as response to several light stimuli. Figure S5. Linear regression fit to scotopic b-wave amplitude at 0.0095 cd.s/m2 and 9.49 cd.s/m2 in WT and 3×Tg-AD mice. Figure S6. OP1 time to peak values at 4, 8, 12 and 16 months of age, based on fERG analysis. Figure S7. Linear regression fit to base wave, first harmonic and second harmonic amplitudes and phases in WT and 3×Tg-AD mice. Figure S8. Spearman correlation between total retinal thickness and grey matter volume of the visual cortex, and photopic flicker response amplitude and grey matter volume of the visual cortex. [file 13195_2019_542_MOESM1_ESM.docx]

**Supplementary figures**


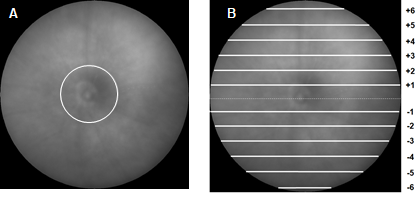


Fig. S1. OCT scan acquisition. (A) One circular scan was acquired around the optic nerve head (ONH), and (B) six linear scans were acquired above and below the ONH. The green lines represent the scans.


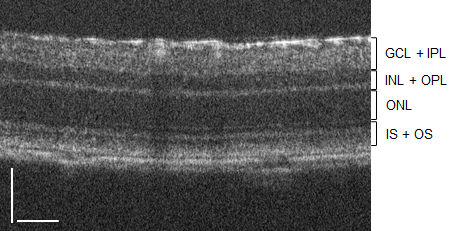


Fig. S2. Retinal layers visualized by OCT. GCL+IPL (ganglion cell layer and inner plexiform layer; INL+OPL (inner nuclear layer and outer plexiform layer); ONL (outer nuclear layer); IS+OS (photoreceptors inner and outer segments. Scale bar: 50 µm.

**
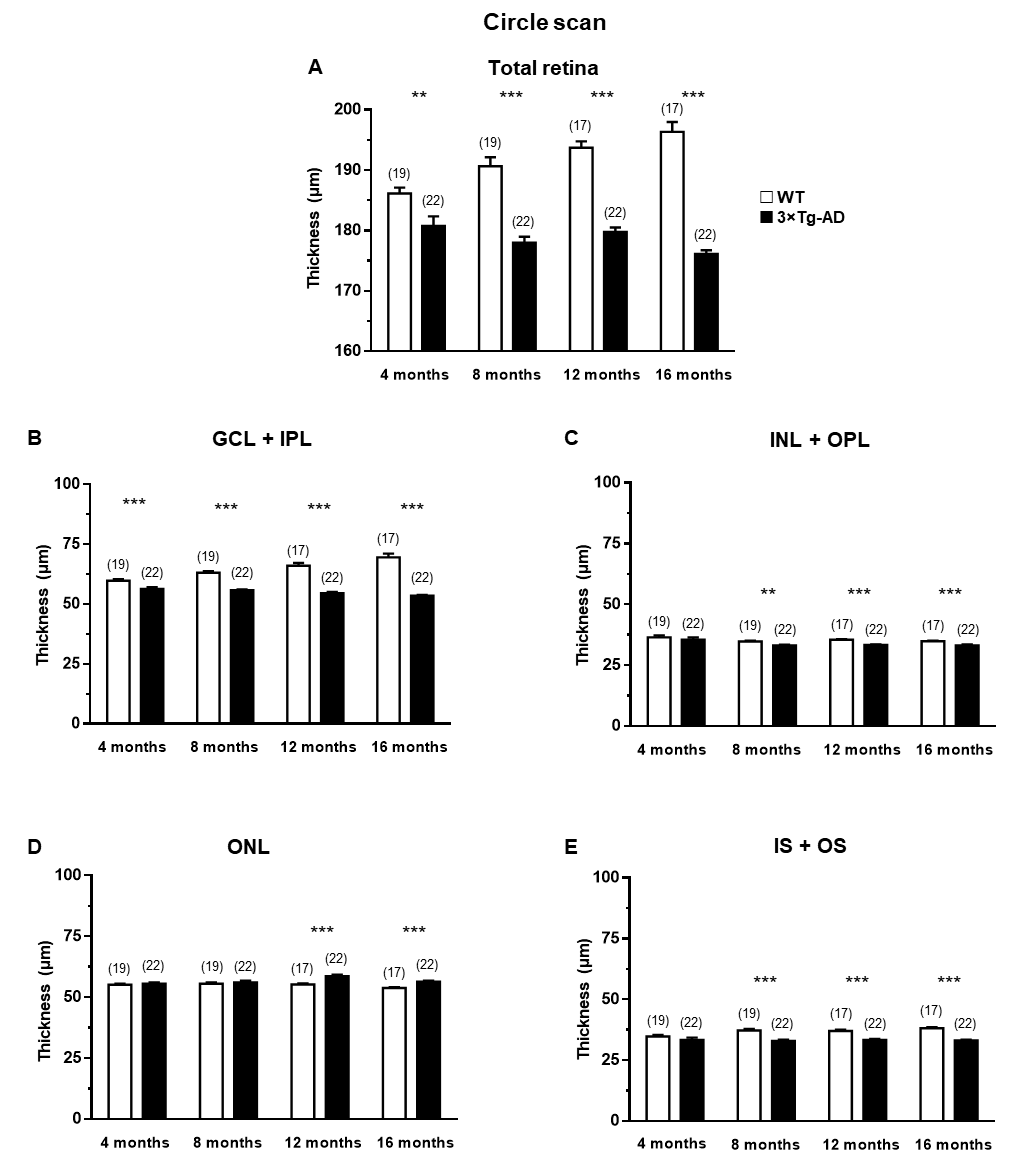
**

**Fig. S3.** Thickness of different retinal layers in WT (white bars) and 3×Tg-AD (black bars) mice at 4, 8, 12 and 16 months of age, based on i*n vivo* OCT circle scans. The thickness of retinal layers was measured using the InSight software. (A) Total retina; (B) GCL+IPL; (C) INL+OPL; (D) ONL; (E) IS+OS. The results are presented as mean ± SEM. ***p*<0.01, ****p*<0.001, according to the Student’s t-test. n _WT_: at 4 months = 19, at 8 months = 19, at 12 months = 17, at 16 months = 17; n _3×Tg-AD_: at 4 months = 22, at 8 months = 22, at 12 months = 22, at 16 months = 22.


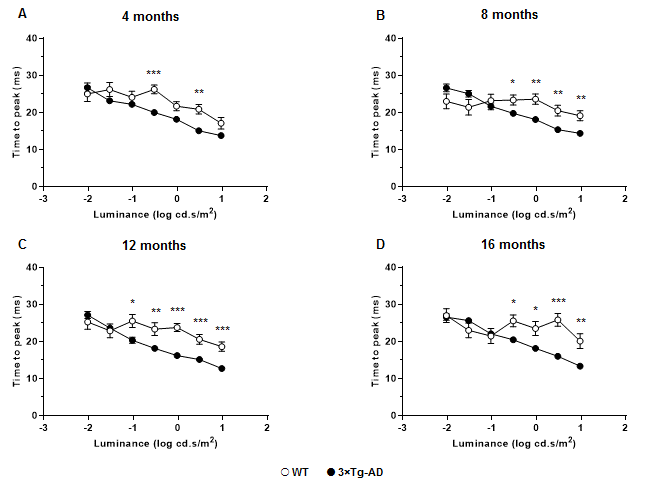


**Fig. S4.** Scotopic *a*-wave time to peak in WT (white circles) and 3×Tg-AD (black circles) mice, at (A) 4, (B) 8, (C) 12 and (D) 16 months of age, as response to several light stimuli. The results are presented as mean ± SEM. **p*<0.05, ***p*<0.01, ****p*<0.001, according to the Student’s t-test. n _WT_: at 4 months = 19, at 8 months = 19, at 12 months = 17, at 16 months = 17; n _3×Tg-AD_: at 4 months = 23, at 8 months = 23, at 12 months = 22, at 16 months = 22.


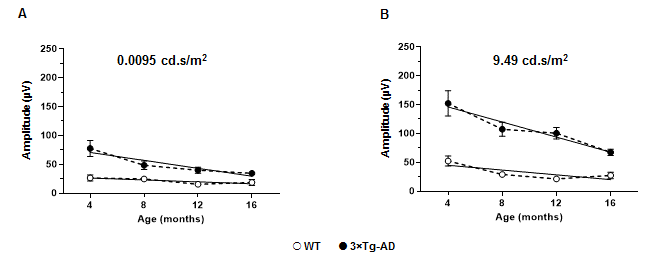


**Fig. S5.** Linear regression fit to scotopic *b*-wave amplitude at (A) 0.0095 cd.s/m^2^ and (B) 9.49 cd.s/m^2^ in WT (white circles) and 3×Tg-AD (black circles) mice. *B*-wave amplitude difference between WT and 3×Tg-AD mice was assessed by comparing slopes and intercepts with ANCOVA; *p*<0.05.


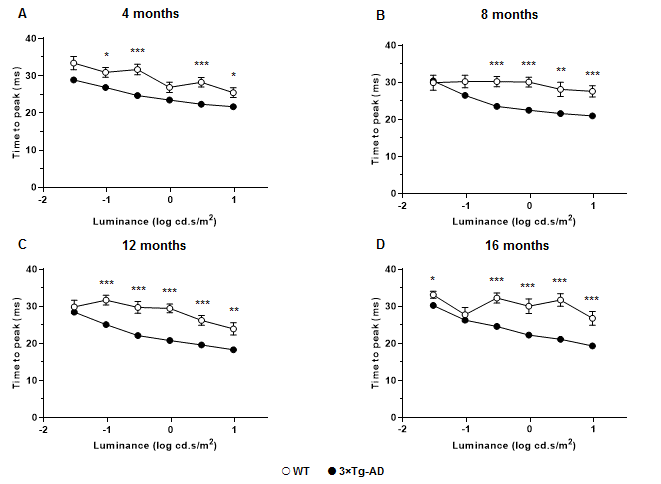


**Fig. S6.** OP1 time to peak values at (A) 4, (B) 8, (C) 12 and (D) 16 months of age, based on fERG analysis. The results are presented as mean ± SEM. **p*<0.05, ***p*<0.01, ****p*<0.001, according to the Student’s t-test. n _WT_: at 4 months = 19, at 8 months = 19, at 12 months = 17, at 16 months = 17; n _3×Tg-AD_: at 4 months = 23, at 8 months = 23, at 12 months = 22, at 16 months = 22.


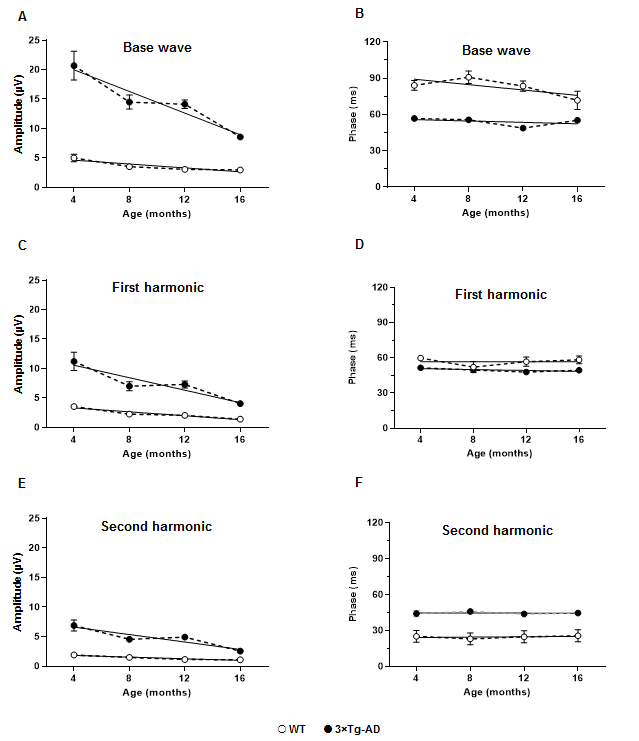


**Fig. S7.** Linear regression fit to (A, B) base wave, (C, D) first harmonic and (E, F) second harmonic amplitudes and phases in WT (white circles) and 3×Tg-AD (black circles) mice. Photopic flicker harmonic amplitude difference between WT and 3×Tg-AD mice was assessed by comparing slopes and intercepts with ANCOVA; *p*<0.05.


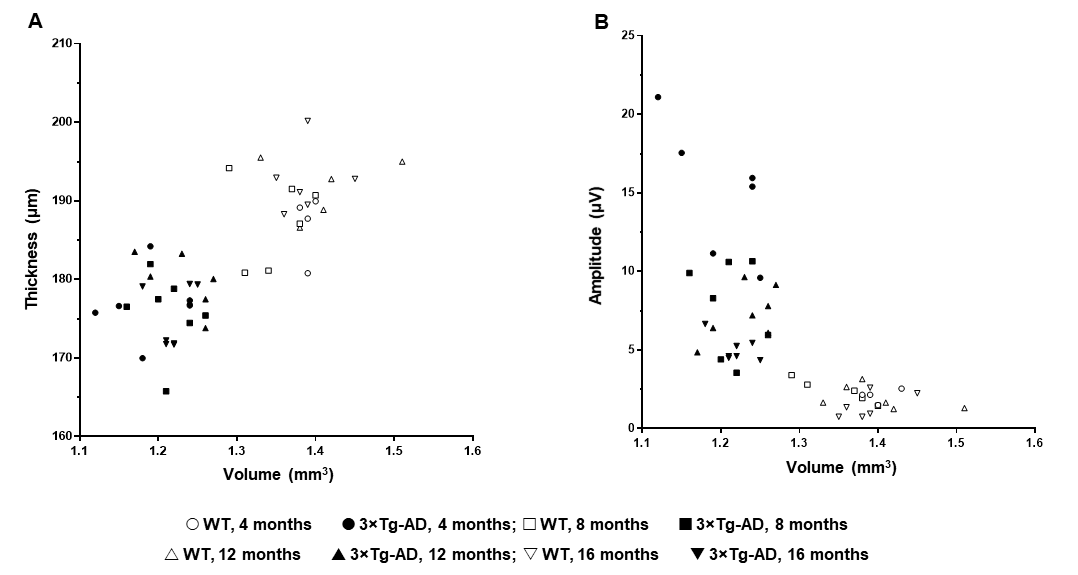


**Fig. S8.** Correlation between (A) total retinal thickness and grey matter volume of the visual cortex, and (B) photopic flicker response amplitude and grey matter volume of the visual cortex, using Spearman correlation. The retinal thickness was measured using OCT line scan. WT and 3×Tg-AD mice were tested at 4 (open and closed circles, respectively), 8 (open and closed squares, respectively), 12 (open and closed up-pointing triangles, respectively) and 16 (open and closed down-pointing triangles, respectively) months of age. n _WT_ = 6, n _3×Tg-AD_ = 7.
